# Comparison of different in vitro differentiation conditions for murine female germline stem cells

**DOI:** 10.1111/cpr.12530

**Published:** 2018-10-17

**Authors:** Kang Zou, Jian Wang, Haiwei Bi, Yabin Zhang, Xueli Tian, Ning Tian, Wanyun Ma, Ji Wu

**Affiliations:** ^1^ Key Laboratory for the Genetics of Developmental & Neuropsychiatric Disorders (Ministry of Education), Bio‐X Institutes Shanghai Jiao Tong University Shanghai China; ^2^ State Key Laboratory of Low‐Dimensional Quantum Physics, Department of Physics Tsinghua University Beijing China; ^3^ Key Laboratory of Fertility Preservation and Maintenance of Ministry of Education Ningxia Medical University Yinchuan China; ^4^ State Key Laboratory of Oncogenes and Related Genes Shanghai Cancer Institute, Renji Hospital, Shanghai Jiao Tong University School of Medicine Shanghai China

**Keywords:** differentiation, female germline stem cells, in vitro, mouse

## Abstract

**Objectives:**

In vitro differentiation of oocytes from female germline stem cells (FGSCs) has exciting potential applications for reproductive medicine. Some researchers have attempted to reveal the in vitro differentiation capacity of FGSCs. However, no systematic comparative study of in vitro differentiation conditions has been performed for murine FGSCs (mFGSCs).

**Materials and Methods:**

mFGSCs line was cultured under five different conditions for in vitro differentiation. RT‐PCR was performed to detect the expression of *Oct4*,* Fragilis*,* Blimp1*,* Mvh*,* Scp3* and *Zp3*. Immunofluorescence was carried out to test the expression of Mvh, Fragilis and Zp3. Two‐photon laser‐scanning microscope was used to analyze nucleus‐plasma ratio, and the proportion of chromatin of GV oocytes differentiated from mFGSCs in vitro (IVD‐GVO), GV oocytes from in vivo (GVO) and mFGSCs.

**Results:**

RT‐PCR and immunofluorescence showed that mFGSC line expressed germ cell‐specific markers, but not a meiosis‐specific marker. By evaluating five different in vitro differentiation conditions, condition 5, which included a hanging drop procedure and co‐culture of mFGSCs with granulosa cells, was shown to be optimal. mFGSCs could be successfully differentiated into germinal vesicle (GV) ‐stage oocytes under this condition. 3D observation revealed that both the nucleus‐plasma ratio and proportion of chromatin were not significantly different between IVD‐GVO and GVO.

**Conclusion:**

We evaluated five in vitro differentiation conditions for mFGSCs and successfully differentiate mFGSCs into GV‐stage oocytes using a three‐step differentiation process.

## 1 INTRODUCTION

Infertility is a reproductive health problem that affects approximately 13%‐18% of couples in the human population.^1^ Affected individuals and their partners suffer from enormous psychological and emotional trauma. It is well known that women produce far fewer gametes than men throughout the course of the reproductive process. Impaired release of fertilizable oocytes is the main cause of female infertility.^2^ As such, precise mechanisms regulating the process of mammalian oogenesis are a longstanding focus of reproductive and developmental biology research.

Recently, researchers have reported the possibility of deriving female gametes from stem cells in vitro.^3^ In 2003, Hubner et al first reported that mouse embryonic stem cells (mESCs) in culture could develop into oogonia that entered meiosis, recruited adjacent cells to form follicle‐like structures, and later developed into blastocysts.^4^ However, Novak et al found that mESC‐derived oocytes did not progress through meiosis.^5^ Interestingly, Lacham‐Kaplan et al showed that male mESCs form ovarian‐like structures containing putative oocytes when cultured in newborn mouse testicular cell‐conditioned medium.^6^ Qing et al found that ovarian granulosa cells induced mESCs to differentiate into oocyte‐like cells, which expressed meiosis‐ and oocyte‐specific genes.^7^ Eguizabal et al achieved complete in vitro differentiation of human induced pluripotent stem cells (iPSCs) into post‐meiotic cells.^8^ Hayashi et al generated primordial germ cell‐like cells (PGCLCs) from mESCs and iPSCs in vitro. After transplantation under mouse ovarian bursa, aggregation of PGCLCs with female gonadal somatic cells generated oocytes exhibiting the capacity for fertilization that results in offspring.^9^, ^10^ In 2016, Hikabe et al reported the reconstitution of the entire mouse oogenesis cycle from ESC‐ and iPSC‐derived PGCLCs completely within a dish.^11^ Although significant breakthroughs have been made to produce female gametes derived from ESCs and iPSCs in vitro, it still remains challenging for reproductive medicine applications. To obtain ESCs, blastocysts must be destroyed, which has been criticized and considered to be unethical. While their origins are less controversial, iPSCs contain exogenous transgenes that increase potential risks for clinical applications.

An important alternative approach for in vitro oogenesis is based on FGSCs, a newly documented germline stem cell present in postnatal mammalian ovaries. Our previous study demonstrated isolation of FGSCs from neonatal and adult mouse ovaries by mouse vasa homology (MVH)‐based magnetic‐activated cell sorting.^12^ FGSCs in long‐term culture maintained their capacity to produce normal oocytes and fertile offspring after transplantation into ovaries.^12^ Wu et al further traced and characterized the development of transplanted FGSCs in vivo.^13^ Xie et al reported similar morphological and molecular signatures between female and male germline stem cells.^14^ Li et al systematically identified and compared the expression profiles of lncRNAs and circRNAs in mFGSCs.^15^ Moreover, Zhang et al performed integrative epigenomic analysis to reveal the unique epigenetic signatures involved in unipotency of mFGSCs.^16^ In 2012, White et al reported ovaries of reproductive‐age women possessed rare mitotically active germ cells that could be propagated in vitro.^17^ Ding et al generated GV‐stage oocytes from human FGSCs obtained from follicular aspirates.^18^ Additionally, Zhou et al obtained FGSCs from female rat ovaries and developed a three‐step system to differentiate rat FGSCs into GV‐stage oocytes in vitro.^19^ Many attempts have been made to reveal the differentiation capacity of FGSCs in vitro and in vivo.^20^, ^21^, ^22^, ^23^, ^24^ However, no systematic comparative study of in vitro differentiation conditions for mFGSCs has been reported.

Therefore, in this study, we evaluated five different in vitro differentiation conditions for mFGSCs. Results showed that a three‐step procedure was the optimal differentiation condition for differentiating mFGSCs into GV‐stage oocytes. Furthermore, we preliminarily studied the characteristics of these in vitro‐differentiated cells using RT‐PCR, fluorescent immunocytochemistry, and two‐photon laser‐scanning microscope (TPLSM). Our study not only provides a tool to systematically identify factors and pathways involved in promoting oogenesis from FGSCs, but also has exciting potential applications for reproductive and regenerative medicine.

## 2 MATERIALS AND METHODS

### 2.1 Animals

Three‐ and six‐week‐old CD‐1 wild‐type female mice were used in this study. All procedures involving animals were approved by the Institutional Animal Care and Use Committee of Shanghai and were conducted in accordance with the National Research Council Guide for Care and Use of Laboratory Animals.

### 2.2 FGSC culture

An FGSC line reported in our previous study was maintained as described.^12^ Briefly, FGSCs were cultured on mitomycin C‐treated (10 μg/mL, Sigma) mitotically inactivated STO cell feeders (derived from mouse SIM embryonic fibroblasts, strain SIM, 5 × 10^4^ cells/cm^2^, ATCC). Culture medium for FGSCs was Minimum Essential Medium α (MEM‐α; Life Technologies) supplemented with 10% foetal bovine serum (FBS; Front), 1 m mol L^−1^ sodium pyruvate (Sigma), 1 m mol L^−1^ non‐essential amino acids (NEAA; Life Technologies), 2 m mol L^−1^ L‐glutamine (Sigma), 0.1 m mol L^−1^ β‐mercaptoethanol (Sigma), 10 ng/mL mouse leukaemia inhibitory factor (LIF; Santa Cruz Biotechnology), 10 ng/mL mouse epidermal growth factor (EGF; PeproTech), 40 ng/mL mouse glial cell line‐derived neurotrophic factor (GDNF; PeproTech), 10 ng/mL human basic fibroblast growth factor (bFGF; PeproTech)and 15 μg/mL penicillin. The medium was changed every 2 days and FGSCs were subcultured every 4‐7 days at a 1:2‐1:3 dilution. Cultures were maintained at 37°C in a 5% CO_2_ atmosphere and cultured for 4‐5 days before in vitro differentiation.

### 2.3 Isolation and culture of mouse granulosa cells

Granulosa cells (GCs) were isolated and cultured as previously described with some modification.^18^, ^25^ Briefly, ovaries from 3‐week‐old CD1 female mice were dissected free of fat, bursa and oviduct. After washing with phosphate‐buffered saline (PBS), GCs were released by manually puncturing ovaries with 25‐gauge needles. Cell suspensions were then passed through a 40‐μm nylon cell strainer and centrifuged at 300 × *g* for 5 minutes. Pelleted cells were reconstituted in Dulbecco's modified Eagle's medium (DMEM; Life Technologies) supplemented with 10% FBS, 1 m mol L^−1^ NEAA and 6 mg/L penicillin. For in vitro differentiation of FGSCs, GCs between the second and fourth passage were treated with mitomycin C (10 mg/mL) for 2‐3 hours, washed in PBS, and then plated in a culture dish pre‐coated with 0.1% (w/v) gelatin.

### 2.4 Preparation of ovarian homogenate

Ovarian homogenate was prepared as previously described with some modification.^26^ Ovaries from 5‐6 wild‐type adult mice were harvested and homogenized in 2 mL of D‐Hanks buffer. Homogenates were subsequently filtered through a 0.22‐μm membrane to remove cell debris, aliquoted and stored at 4°C for further use.

### 2.5 In vitro differentiation

First, STO feeder cells were removed from FGSC cultures by differential adherence. Briefly, cells were trypsinized and plated on a 0.1% (w/v) gelatin‐coated culture dish. After 30 minutes, most STO cells had attached to the dish. Non‐adherent cells were collected to perform in vitro differentiation. Five differentiation conditions were evaluated. Detailed compositions of each differentiation medium are shown in Table 1. All cultures were maintained at 37°C in a 5% CO_2_ atmosphere with morphological features monitored daily.

**Table 1:** The differentiation media for mouse female germline stem cells

	Medium 1	Medium 2	Medium 3	Medium 4	Medium 5a	Medium 5b	Medium 5c
FBS (%)	10	10	10	10	10	10	15
NEAA (m mol L^−1^)	1	1	1	1	1	1	1
L‐Glu (m mol L^−1^)	2	2	2	2	2	2	2
Sodium pyruvate (m mol L^−1^)	1	1	1	1	1	1	1
β‐mercaptoethanol (m mol L^−1^)	0.1	0.1	0.1	0.1	0.1	0.1	0.1
Penicillin (μg/mL)	15	15	15	15	15	15	15
LIF	—	—	—	—	—	—	—
bFGF (ng/mL)	20	20	20	20	—	10	10
EGF (ng/mL)	10	10	10	—	—	—	10
GDNF (ng/mL)	10	10	10	—	—	—	—
BMP4 (ng/mL)	—	—	—	—	—	10	—
RA (M)	10^−7^	—	10^−7^	—	—	10^−7^	—
Ovarian homogenate (μL)	—	100	100	—	—	—	100
Oestrogen (ng/mL)	—	—	—	1	—	—	1
Progesterone (ng/mL)	—	—	—	1	—	—	1
PMSG (IU/mL)	—	—	—	—	—	—	1
hCG (IU/mL)	—	—	—	—	—	—	1
Transferrin (μg/mL)	—	—	—	—	—	—	5
Insulin (μg/mL)	—	—	—	—	—	—	10
MEM‐α	Up to 10 mL	Up to 10 mL	Up to 10 mL	Up to 10 mL	Up to 10 mL	Up to 10 mL	Up to 10 mL
Granulosa cells	—	—	—	—	—	+	+
Hanging drop	—	—	—	—	+	—	—

Condition 1: Non‐adherent cells were plated on 0.1% (w/v) gelatin‐coated non‐feeder wells of a 24‐well plate and cultured in LIF‐withdrawal, but RA added, differentiation medium (Medium 1).

Condition 2: Non‐adherent cells were plated on 0.1% (w/v) gelatin‐coated non‐feeder wells of a 24‐well plate and cultured in LIF‐withdrawal, but ovarian homogenate was added to the differentiation medium (Medium 2).

Condition 3: Non‐adherent cells were plated on 0.1% (w/v) gelatin‐coated non‐feeder wells of a 24‐well plate and cultured in LIF‐withdrawal, but RA and ovarian homogenate were added to the differentiation medium (Medium 3).

Condition 4: Non‐adherent cells were plated on 0.1% (w/v) gelatin‐coated non‐feeder wells of a 24‐well plate and cultured in Medium 3 for 10 days. Next, cells were cultured in medium supplied with oestrogen (1 ng/mL) and progesterone (1 ng/mL) for 10 days (Medium 4).

Condition 5: Stage 1: Non‐adherent cells were collected in culture medium without growth factors (Medium 5a). Next, cells were placed under the lid of a petri dish containing 3 mL of PBS and cultured in hanging drops at 20 μL per drop for 2 days. Stage 2: After 2 days, cells were collected and resuspended in medium containing 10 ng/mL bFGF (PeproTech), 10 ng/mL bone morphogenic protein (BMP)‐4 (PeproTech) and 0.1 μ mol L^−1^ RA (Sigma) (Medium 5b), and then cultured on a mitomycin C‐treated granulosa cell monolayer for 4‐5 days. The medium was changed every 2 days. Stage 3: Finally, cells were cultured in differentiation Medium 5c containing EGF (10 ng/mL), bFGF (10 ng/mL), transferrin (5 mg/mL), insulin (10 mg/mL), pregnant mare serum gonadotrophin (PMSG; 1 IU/mL), hCG (1 IU/mL), oestrogen (1 ng/mL), progesterone (1 ng/mL) and ovarian homogenate. The medium was changed every 2 days. Every 6 days, cells were transferred onto a freshly treated granulosa cell monolayer. This process was performed for 20‐25 days.

### 2.6 RT‐PCR analysis

Total RNA from cells was prepared using a PicoPure RNA Isolation Kit (Thermo Fisher Scientific) according to the manufacturer's instructions. Reverse transcription was performed using a HiScript® II Q RT Super Mix (+ gDNA wiper) Kit (Vazyme) following the manufacturer's protocol. For RT‐PCR, 35 cycles of PCR were performed using Taq MasterMix (Dye) (CWBIO) with primer sets specific for *Oct4*,* Fragilis*,* Blimp1*,* Mvh*,* Scp3*,* Zp3* and *Gapdh*.

### 2.7 BrdU labelling

BrdU (50 mg/mL; Sigma) was supplied into FGSCs culture medium to a final concentration of 50 mg/mL, and FGSCs was incubated for 5 hours with BrdU‐containing medium before immunofluorescence assay.

### 2.8 TUNEL staining

The protocol for TdT‐mediated dUTP **N**ick‐End Labelling (TUNEL) staining was performed based on a TdT‐mediated dUTP **N**ick‐End Labelling kit (Beyotime, China). Briefly, FGSCs after differentiation treatment were plated on poly‐lysine coated 96‐well dishes for 1 hour. Subsequently, the cells were fixed with 4% PFA and treated with 0.3% triton X‐100 for permeation. After rinse with PBS, TdT enzyme contained TUNEL fluorescent staining medium was supplied to samples, and the samples were placed in the dark for 1 hour. Then, the medium was aspirated, and the samples were rinsed with PBS for twice, and DAPI was added for counterstaining. Finally, observed the sample under fluorescent microscope.

### 2.9 Immunofluorescence

Culture media were discarded gently, and cells were washed carefully with PBS, followed by fixation using 4% paraformaldehyde at room temperature for 15‐20 minutes. After rinsing twice with PBS, cells were blocked using 10% goat serum in a humid box at 37°C for 15 minutes. Cells were then incubated with primary antibodies diluted in PBS [BrdU (1:200; Chemicon), Mvh (1:200; Abcam), Fragilis (1:200; Abcam), Zp3 (1:200; Abcam)] in a humid box at 37°C for 1 hour. After washing with PBS, 1:150 PBS‐diluted goat anti‐rabbit IgG labelled with TRITC or FITC was applied to cells and incubated at 37°C for 40 minutes. Images were obtained with a Leica DM2500 microscope and Leica DFC 550 digital camera.

### 2.10 Fluorescence staining and image acquisition

Female CD‐1 mice aged 6 weeks were sacrificed by cervical dislocation, and their ovaries were removed and transferred to PBS. Oocytes were released by puncturing the ovarian follicles with a needle. Denuded GV oocytes were selected for controls. At the same time, mFGSCs and in vitro differentiated FGSCs under condition 5 were collected by mouth pipette. The three cell types were immediately fixed in 4% paraformaldehyde for 30 minutes at room temperature. Fixed cells were washed three times in PBS before staining nuclei with 5 µg/mL Hoechst 33342 for 15 minutes at 37ºC. Following three washes in PBS, GV oocytes, mFGSCs and in vitro‐differentiated FGSCs were placed on glass‐bottom dishes (MatTek Corp.) for observation with a two‐photon laser‐scanning microscope (TPLSM).

### 2.11 Image analysis

Consecutive optically sectioned images of GV oocytes, in vitro‐differentiated FGSCs, and mFGSCs were preprocessed and analyzed using 3D reconstruction software Amira 5.2 (Visage Imaging, Berlin, Germany), as previously described.^27^ Critical morphological feature parameters for the three types of cells included cell volume, nucleus volume and chromatin volume, with the coordinates of nuclear and chromatin centres quantified by Amira 5.2.

### 2.12 Statistics

All data are presented as mean ± SEM. Statistical tests were performed with Student's *t* test using Statistical Package for the Social Sciences (SPSS) software (version 20.0; IBM). *P* < 0.05 was considered statistically significant, and *P* < 0.01 was considered highly significant. Graph generation was carried out using SigmaPlot software (version 13.0).

## 3 RESULTS

### 3.1 Characterization of mFGSC line

**Figure 1:**
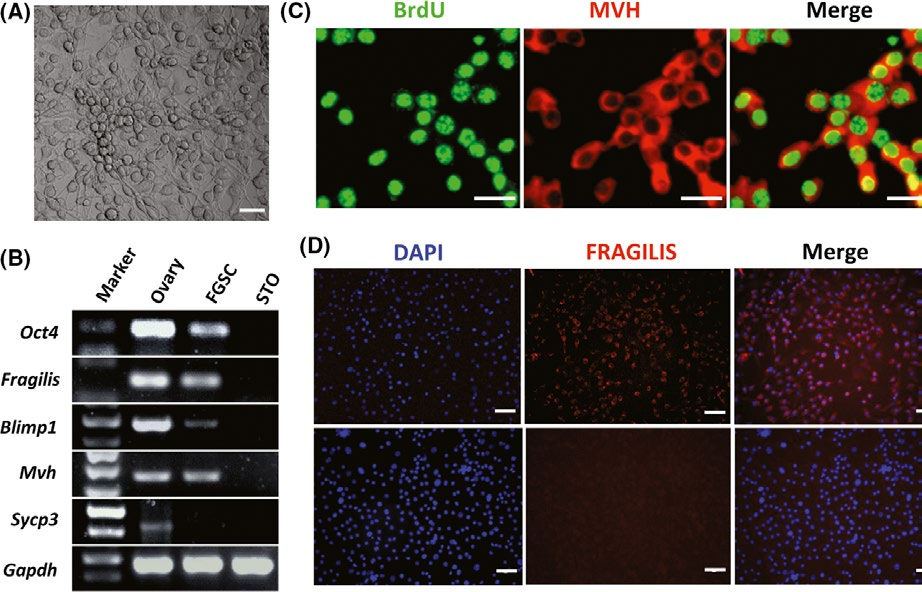
Characterization of mFGSC line. A, A representative morphology of mFGSC line. Scale bar: 40 μm. B, Gene expression profile of mFGSC line. C, Dual immunofluorescence for BrdU (green) and mouse vasa homology (red) in mFGSCs. Scale bars: 20 μm. D, Immunofluorescence staining of mFGSCs for FRAGILIS. Scale bars: 40 μm

The mFGSC line reported in our previous study was subjected to in vitro‐induced differentiation (Figure 1A). After STO feeder cells were removed from mFGSC cultures by differential adherence,and the molecular signatures of the cultured mFGSC line were characterized by examining the expression of genes associated with germline development. RT‐PCR results showed that the cells expressed germ cell‐specific markers (*Mvh*,* Fragilis*,* Blimp1* and *Oct4*), but not a meiosis‐specific marker (*Sycp3*) (Figure 1B). We further confirmed the proliferative potential and germline‐specific protein expression of these cells by immunofluorescence. Immunocytochemical analysis indicated that mFGSCs were positive for germline‐specific markers (Mvh and Fragilis) and BrdU (Figure 1C,D). All characteristics of the mFGSC line detected in this study were consistent with previously reported observations.^12^, ^13^, ^16^

### 3.2 Characteristics of mFGSCs after in vitro differentiation using condition 1

**Figure 2:**
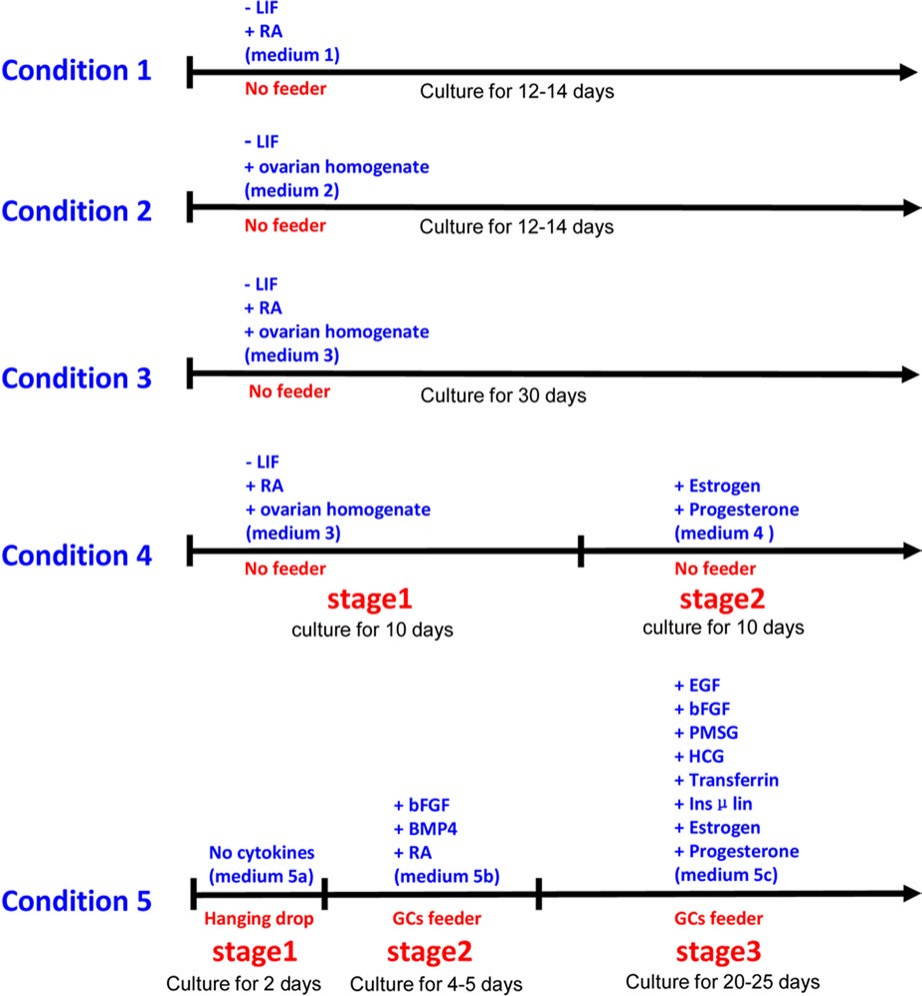
Schematic of mFGSC in vitro differentiation

**Figure 3:**
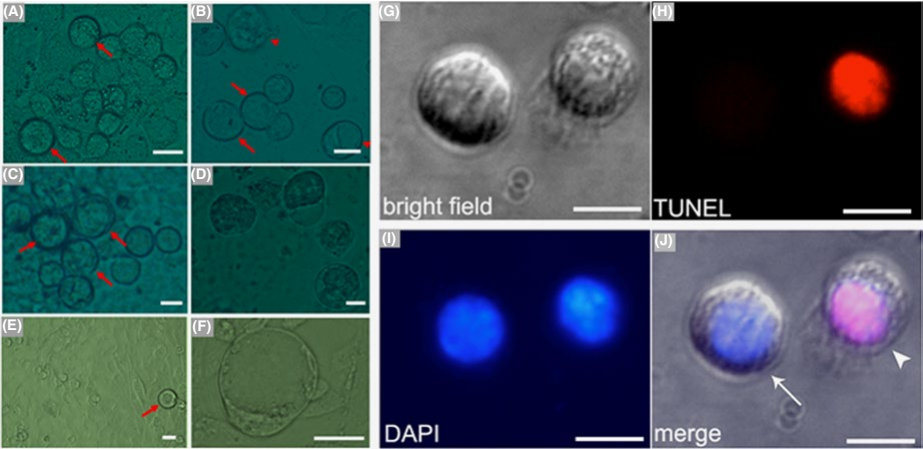
Characteristics of in vitro‐differentiated mFGSCs under conditions 1 and 2. (A‐D) Representative morphological characteristics of mFGSCs after in vitro differentiation with condition 1. Scale bars: 20 μm. A, Morphology of cells at day 2. Spherical cells with large diameters are indicated by red arrows. B, Morphology of cells at day 4. Spherical cells with a diameter greater than 25 μm are indicated by red arrows. Apoptotic cells are indicated by red arrowheads. C, Morphology of cells at day 6. Spherical cells with a diameter of up to nearly 40 μm are indicated by red arrows. D, Morphology of cells at day 8. (E‐F) Representative morphological characteristics of mFGSCs after in vitro differentiation with condition 2. Scale bars: 20 μm. E, Morphology of cells at day 6. Spherical cell enlarged to 30 μm is indicated by red arrow. F, Morphology of “bubble‐like” vacuolar cell at day 8. (G‐J)TUNEL staining in mFGSCs cultured under conditions 1 for 4 days. Scale bars: 20 μm. G, Morphology of cells at day 4. H, TUNEL staining of apoptotic cells. (I) DAPI staining of cells. J, Merged image of (H) and (I). Arrowhead indicates apoptotic cell. Arrow indicates differentiated female germ cell

To differentiate mFGSCs into oocytes in vitro, five differentiation conditions were evaluated (Figure 2). It is well recognized that retinoic acid (RA), produced by the mesonephros of both sexes, causes germ cells in the ovary to enter meiosis and initiate oogenesis.^27^ Thus, after STO feeder cells were removed, the mFGSC line was first exposed to differentiation Medium 1, which was LIF‐withdrawal but RA supplied. There was no significant morphological change after 2 days culture. A minority of round cells exhibited a slight increase in cell diameter (approximately 2‐3 cells per 40× field) to 20‐25 μm (Figure 3A). After 4 days of culture, the diameter of most round cells reached above 25 μm, with approximately one‐third of round cells exhibiting vacuolization and commitment to apoptosis (Figure 3B,G‐J). When cultured for 6 days, round cells with diameters up to nearly 40 μm were observed (Figure 3C). At day 8, the diameter of the majority of cells became significantly enlarged, and these cells soon committed to apoptosis (Figure 3D). mFGSCs gradually became morphologically similar to oocytes with characteristic large diameters when in vitro differentiated in RA‐supplied medium. However, differentiated cells frequently committed to apoptosis. These results suggested that RA could induce differentiation of mFGSCs.

### 3.3 Characteristics of mFGSCs after in vitro differentiation using condition 2

Ovaries, the female reproductive organ, are the site of production and periodical release of egg cells. To evaluate the effect of ovarian homogenate on in vitro differentiation of mFGSCs, mFGSCs were cultured in ovarian homogenate (OH)‐supplied differentiation Medium 2. When conditioned with OH, mFGSCs exhibited better cellular growth behavior. There were only scarce round cells, approximately one per 40× field, which enlarged to 30 μm in diameter after 6 days of culture (Figure 3E). At day 8, a small number of round, “bubble‐like” vacuolar cells were observed (Figure 3F). This culture condition might not be suitable for maintaining survival of differentiating cells. Further morphological changes were not observed in prolonged culture. These observations suggested that OH may not contribute much to the in vitro differentiation of mFGSCs, but it may have a positive effect on the in vitro growth of mFGSCs.

### 3.4 Characteristics of mFGSCs after in vitro differentiation using condition 3

**Figure 4:**
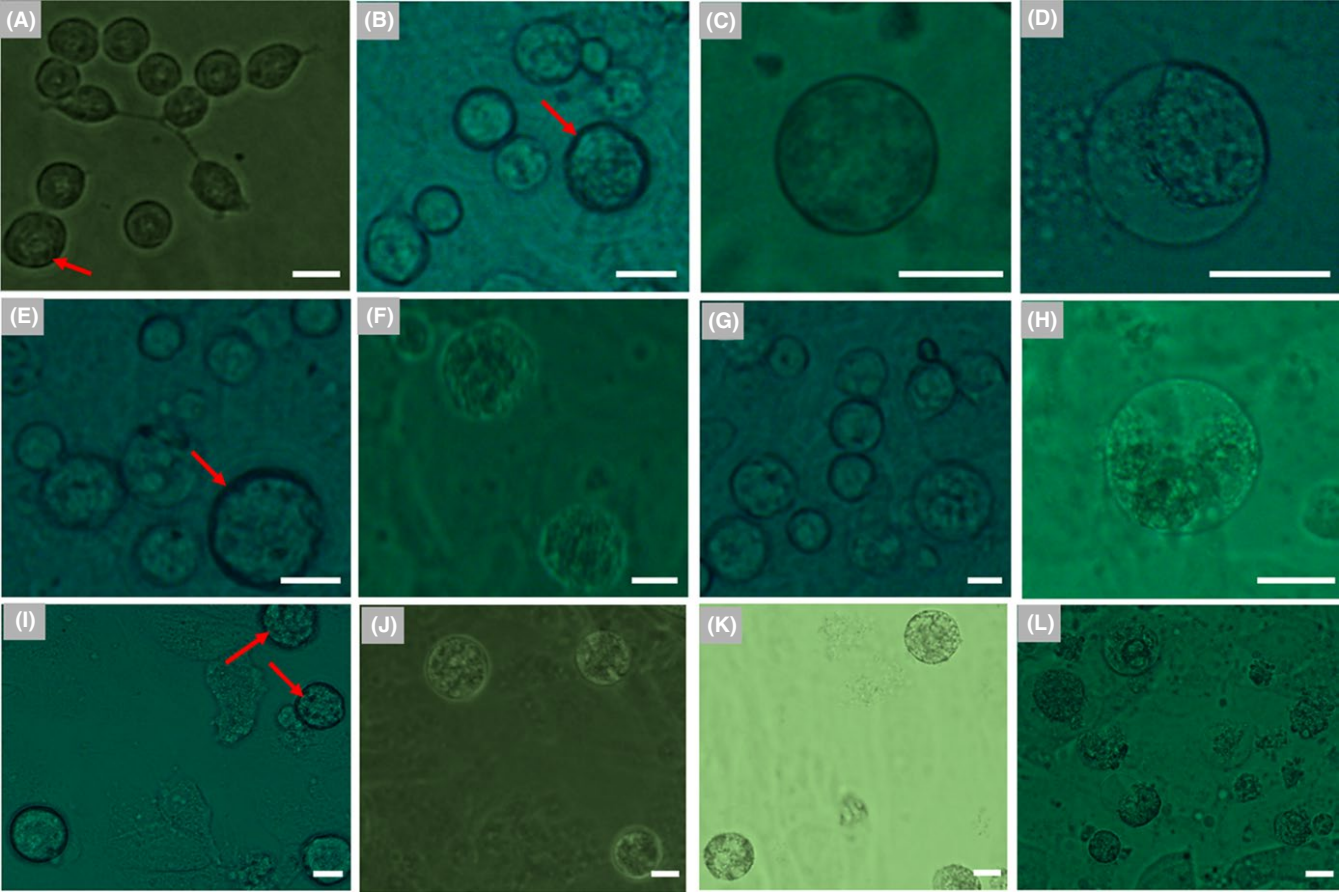
Characteristics of in vitro‐differentiated mFGSCs under condition 3. (A‐L) Representative morphological characteristics of mFGSCs after in vitro differentiation with condition 3. Scale bars: 20 μm. A, Morphology of cells at day 2. Spherical cell with diameter of nearly 25 μm is indicated by red arrow. B, Morphology of cells at day 4. Spherical cell with large diameter is indicated by red arrow. C, Representative morphology of a cell with a 30‐μm diameter at day 4. D, Representative morphology of vacuolar cell at day 4. E, Morphology of cells at day 6. Further enlarged spherical cell is indicated by red arrow. F, Representative morphology of apoptotic cell at day 6. G, Morphology of cells at day 8. H, Representative morphology of a vacuolar cell at day 10. I, Morphology of cells at day 15. Apoptotic cells are indicated by red arrows. J, Morphology of cells at day 20. K, Representative morphology of apoptotic cells at day 20. L, Morphology of cells at day 30

Based on the above observations, RA contributed to the differentiation of mFGSCs, and OH was conducive to maintaining the in vitro survival of mFGSCs. In condition 3, mFGSCs were subjected to in vitro differentiation in Medium 3, which was supplied with both RA and OH. The morphology of mFGSCs remained similar to those induced with RA only for the first 2 days. There were 2‐3 round cells per 40× field observed to be up to 25 μm in diameter (Figure 4A). At day 4, the majority of cells did not show discernible morphological differences compared with cells at day 2 (Figure 4B). Nevertheless, a limited fraction of round cells, one per 2‐3 40× fields, was enlarged to approximately 30 μm in diameter (Figure 4C), with a few cells showing vacuolar morphology suggestive of cell apoptosis (Figure 4D). At day 6, a portion of cells, whose diameters were 30 μm at day 4, further enlarged to 35‐40 μm in diameter, which constituted less than 10% of the total cell count (Figure 4E). Along with these enlarged cells, some cells with diameters of nearly 40 μm were committed to apoptosis (Figure 4F). After 8 days of culture, an increasing number of cells significantly enlarged their size, with a considerable portion being up to 40 μm in diameter (Figure 4G). At day 10, some round cells underwent apoptosis to form vacuoles (Figure 4H).

At day 15, morphological characteristics of cells were similar to those observed at day 10. However, cell number was decreased, and larger cells gradually underwent apoptosis (Figure 4I). When culture was prolonged to 20 days, the majority of cells enlarged to over 30 μm in diameter (Figure 4J), with a considerable number of cells undergoing apoptosis (Figure 4K). At day 30 of culture, almost all cells were committed to apoptosis (Figure 4L).

### 3.5 Characteristics of mFGSCs after in vitro differentiation with condition 4

**Figure 5:**
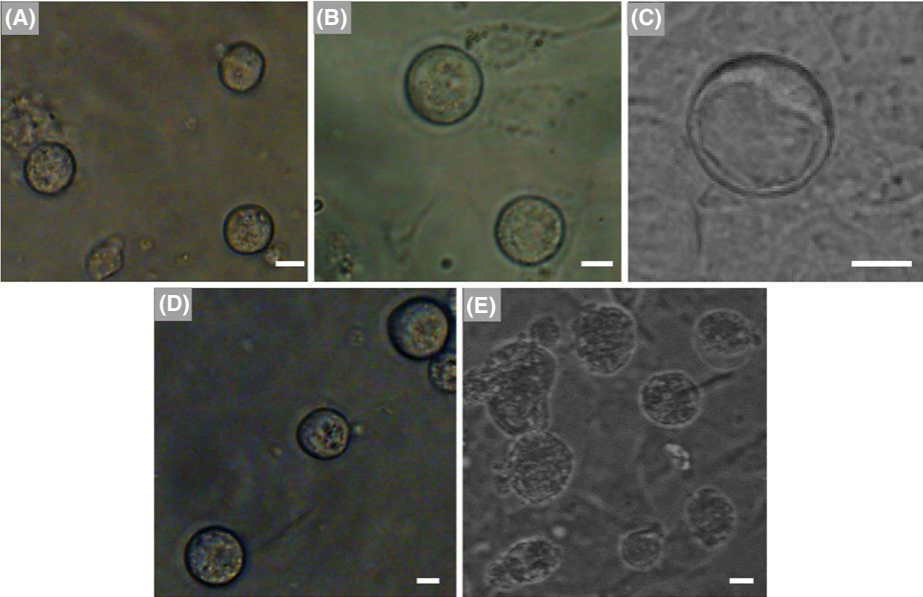
Characteristics of in vitro‐differentiated mFGSCs under condition 4. (A‐E) Representative morphological characteristics of mFGSCs after in vitro differentiation with condition 4. Scale bars: 20 μm. A, Morphology of cells at day 1. B, Representative morphology of cells at day 3. C, Representative morphology of vacuolar cell at day 3. D, Morphology of cells at day 5. E, Morphology of cells at day 7

Oestrogen and progesterone are important endocrine hormones that regulate the development of oocytes.^28^ To evaluate the impact of hormones on differentiating mFGSCs, after 10 days of culture in differentiation Medium 3, cells were exposed to differentiation Medium 4 supplied with oestrogen and progesterone. The first day in Medium 4, no obvious morphological changes were observed (Figure 5A). Two days later, the diameter of some round cells grews to nearly 45 μm (Figure 5B), meanwhile some cells began to form vacuoles (Figure 5C). At day 5, the size of round cells was mostly unchanged (Figure 5D). On day 7, many round cells exhibited severe apoptosis (Figure 5E). Differentiating cells in this condition were prone to apoptosis and could not be maintained longer in culture.

### 3.6 Characteristics of mFGSCs after in vitro differentiation with condition 5

Granulosa cells transduce multiple signals to participate in the initiation of oocyte meiosis and maturation of oocytes through gap junctions and paracrine mechanisms. Moreover, hanging drop is a widely used method for stem cell differentiation, which promotes the aggregated cells to differentiate. Based on the information above and previous studies,^18^, ^19^ we further optimized the in vitro differentiation conditions for mFGSCs using a three‐stage differentiation process (see Materials and Methods). After culture in differentiation Medium 5a in the form of hanging drops for two days, cells were co‐cultured with mitomycin C‐treated granulosa cells in differentiation Medium 5b. When cultured in Medium 5b for 2 days, no obvious morphological variation was observed, except for a few of round cells with larger size (Figure 7A). At day 3, the diameter of a small number of round cells increased to 20‐25 μm (Figure 7B). At day 4, the tendency of differentiation was still not obvious, but a few of round cells enlarged to approximately 30 μm in diameter (Figure 7C). At the same time, some cells began to undergo apoptosis (Figure 7D). At day 6, cells were transferred to differentiation Medium 5c. As differentiation continued, the cells became enlarged. At day 5 in differentiation Medium 5c, round cells grew to 40‐45 μm in diameter and began to form zona pellucida (Figure 7E). When cells were differentiated in Medium 5c for 11‐18 days, many round cells continued to grow and exhibited diameters enlarged to more than 50 μm (Figure 7F). After culture to day 18, a few round cells kept developing and the structures of germinal vesicle and zona pellucida were also observed (Figure 7G‐J). These results suggested that this condition was optimal for in vitro differentiation of mFGSCs.

### 3.7 Expression analysis of Scp3 and Zp3

**Figure 6:**
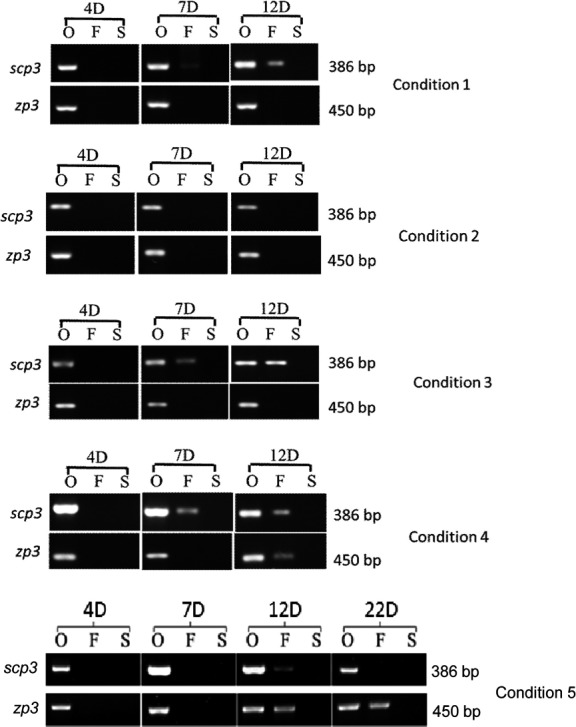
Expression of Scp3 and Zp3 in in vitro‐differentiated mFGSCs under differentiation condition 1‐5 at day 4, 7, 12 and 22 (only for condition 5). O: ovary; F: in vitro‐differentiated mFGSCs; S: STO; D: days

**Figure 7:**
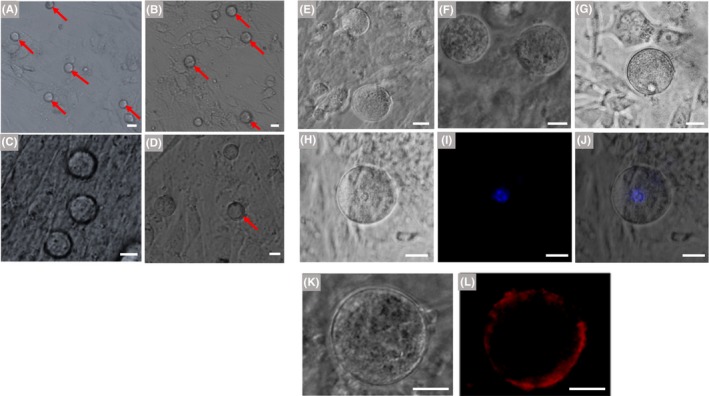
Characteristics of in vitro‐differentiated mFGSCs under condition 5. (A‐D) Representative morphology of cells in differentiation Medium 5b at day 2, 3 and 4, respectively. A, Morphology of cells at day 2. Round cells with larger sizes are indicated by red arrows. Scale bar: 20 μm. B, Morphology of cells at day 3. Spherical cells with a diameter of 20‐25 μm are indicated by red arrows. Scale bar: 20 μm. C, Morphology of cells at day 4. Scale bar: 20 μm. D, Some cells exhibited apoptosis at day 4. Red arrow indicates apoptotic cell. Scale bar: 20 μm. E, Representative morphology of cells in differentiation Medium 5c at day 5. Scale bar: 20 μm. F, Morphology of cells in differentiation Medium 5c at day 11. Scale bar: 20 μm. G, Morphology of cells in differentiation Medium 5c at day 18. Scale bars: 20 μm. (H‐J) An example of in vitro‐differentiated mFGSCs with germinal vesicle. Nuclei were stained with Hoechst 33342. (H) Bright field, (I) Hoechst 33342 and (J) merged images. Scale bars: 20 μm. (K, L): Immunofluorescence analysis of ZP3 in in vitro‐differentiated mFGSCs. (K) Bright field and (L) immunofluorescence images. Scale bars: 20 μm

The developmental stages of cells during differentiation in these conditions were examined. Expression of *Scp3* (a meiosis‐specific marker) and *Zp3* (an oocyte‐specific marker) was detected at days 4, 7, 12 and 22 of differentiation. For condition 1‐4, expression of Sycp3 was detected as early as day 7 under condition 1, 3 and 4, while it was not detected under condition 2 (Figure 6). Moreover, expression of Zp3 was only detected at day 12 under condition 4, indicating that condition 4 is more conducive to FGSCs differentiation (Figure 6). For condition 5, the results of RT‐PCR showed that both *Scp3* and *Zp3* were expressed at day 12. At day 22 of culture, *Zp3* was still expressed, whereas *Scp3* expression could not be detected (Figure 7K). Expression of *Scp3* suggested that cells could be committed to meiosis, while the *Zp3* expression indicated that cells could form zona pellucida. Based on RT‐PCR results, formation of zona pellucida in cells was further confirmed by immunocytochemical analysis with an antibody against Zp3. Zp3 expression was detected in cells with diameters of approximately 60 μm (Figure 7L,M).

### 3.8 3D observation and preliminarily quantitative analysis of GV oocytes differentiated from mFGSCs in vitro, GV oocytes from in vivo and mFGSCs

**Figure 8:**
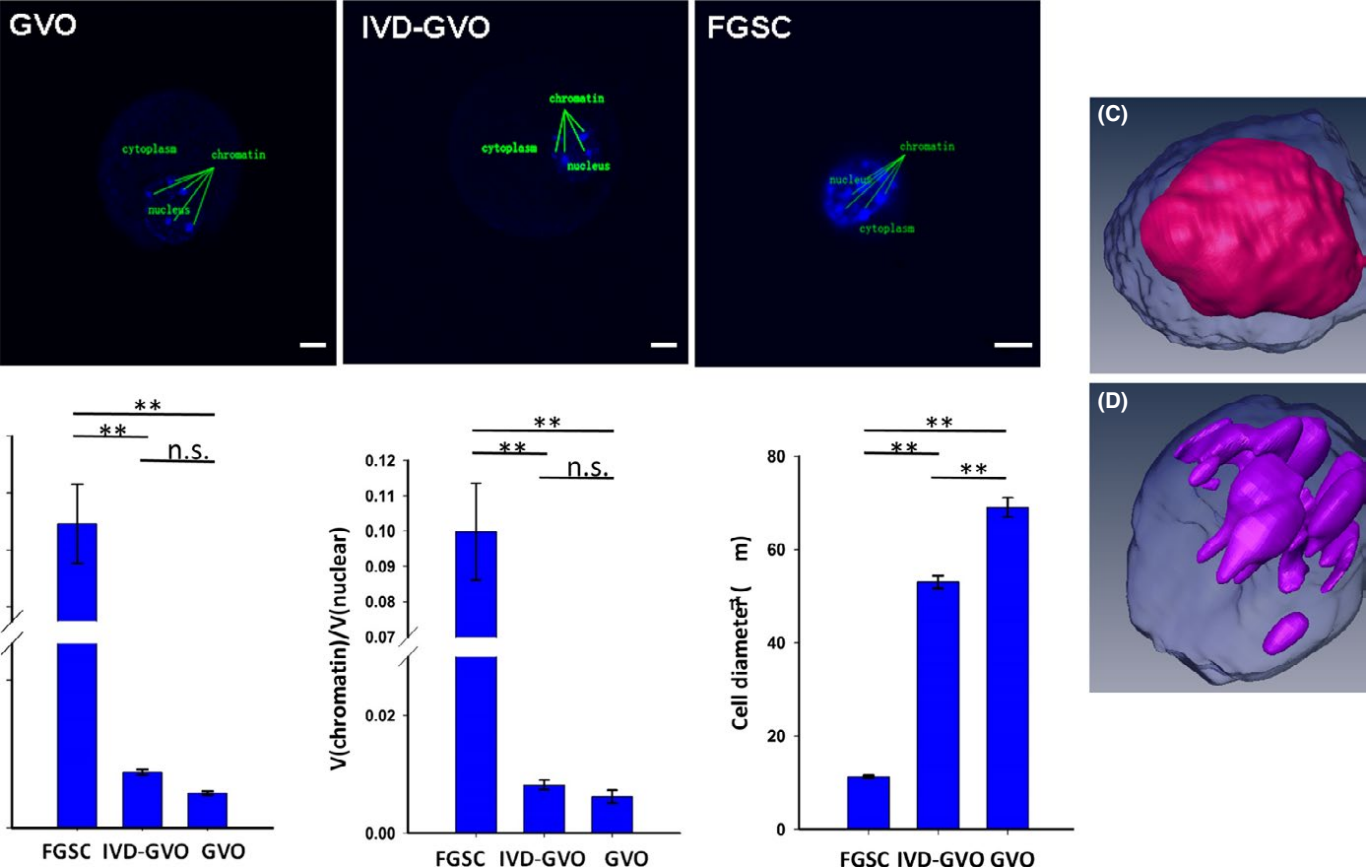
Three‐dimensional observation of GV oocytes differentiated from mFGSCs in vitro, GV oocytes from in vivo, and mFGSCs. A, X‐Y projection (top view) of the z‐axis of GV oocytes differentiated from mFGSCs in vitro (IVD‐GVO), GV oocytes from in vivo (GVO) and mFGSCs as observed by TPLSM. Nuclei were stained with Hoechst 33342. Scale bars: 10 μm (GVO and IVD‐GVO), 5 μm (FGSC). B, Statistical analysis of cell diameter, the nuclear‐to‐cytoplasm ratio [V(nuclear)/V(cytoplasm)] and proportion of chromatin [V(chromatin)/V(nuclear)] of GVO, IVD‐GVO and mFGSCs. **Indicates *P* < 0.01. No significant difference (ns) indicates *P* > 0.05. n ≥ 5 per group. (C‐D) Reconstructed three‐dimensional images mFGSC using Amira software. C, Nuclear distribution of mFGSCs. The blue background refers to cytoplasm, and the pink shape indicates nucleus. D, Chromatin distribution of mFGSCs. The blue background refers to the nucleus, while the purple shape indicates chromatin

To further study the characteristics of in vitro‐differentiated mFGSCs under condition 5, three types of cells including GV oocytes differentiated from mFGSCs in vitro (IVD‐GVO), GV oocytes from in vivo (GVO), and mFGSCs, were collected for 3D observation by TPLSM. Images of the x‐y plane for these three types of cells are shown in Figure 8A. There was a significant difference in diameter between IVD‐GVO (53 ± 1.39 μm) and GVO (69 ± 2.09 μm), *P* < 0.01). However, the diameter of both IVD‐GVO and GVO was much larger than mFGSCs (11.2569 ± 0.31 μm, *P* < 0.01) (Figure 8B). The nucleus‐plasma ratio [V(nuclear)/V(cytoplasm)] of IVD‐GVO was 0.0759 ± 0.0038, which was similar to that of GVO (0.0475 ± 0.0029) (*P* > 0.05). However, mFGSCs exhibited a significantly increased nucleus‐plasma ratio (1.2922 ± 0.1388, *P* < 0.01) compared with either IVD‐GVO or GVO (Figure 8B). Moreover, the proportion of chromatin [V(chromatin)/V(nuclear)] of IVD‐GVO (0.0082 ± 0.0008) was comparable to that of GVO (0.0062 ± 0.0011), but was much smaller than mFGSCs (0.0998 ± 0.0137, *P* < 0.01) (Figure 8B). 3D‐reconstructed images of mFGSCs further demonstrated its larger nucleus‐plasma ratio and proportion of chromatin (Figure 8C,D). These results indicated that all the three parameters of IVD‐GVO resemble those of GVO, but are distinct from mFGSCs.

## 4 DISCUSSION

Successfully obtaining functional oocytes in vitro from stem cells is not only conducive to understanding the regulatory mechanisms of oogenesis, but also improving the fecundity of mammalian females. FGSCs are capable of producing fully functional oocytes and fertile offspring after transplantation into ovaries.^12^, ^13^, ^19^, ^29^ They provide an alternative strategy to study mammalian oogenesis in vitro because of their germline characteristics. In addition, they may provide a valuable model for identifying factors involved in germ cell formation and differentiation.

We preliminarily evaluated five different in vitro differentiation conditions for mFGSCs. It is believed that retinoic acid (RA) causes germ cells in the ovary to enter meiosis and initiates oogenesis.^27^, ^30^ Thus, RA induction was first attempted to promote FGSC differentiation in our study (condition 1). As shown in the results, heterogeneous cultures were observed after supplying RA to differentiation medium. A significant fraction of cells showed differentiating oocyte‐like morphological characteristics with enlarged diameter, suggesting mFGSCs could readily commit to differentiation in the presence of RA. Consistent with in vivo studies demonstrating the requirement of RA for oogenesis, mFGSCs responded well to RA induction in vitro, indicating that our mFGSC line maintains similar differentiation characteristics to its in vivo counterparts. Additionally, it is well recognized that leukaemia inhibitory factor (LIF) is essential for maintaining the undifferentiated state of mouse embryonic stem cells.^31^ Previous reports demonstrated that feeder cells play a crucial role in maintaining the undifferentiated state of FGSCs.^17^, ^18^ Therefore, withdrawing LIF and STO feeders in differentiation condition 1 may further facilitate the differentiation of mFGSCs. However, the resulting differentiated cells were difficult to maintain and prone to apoptosis, suggesting that in vitro differentiation requires more complicated conditions.

Stem cells located within a specific microenvironment, known as a niche that has both anatomical and functional dimensions.^32^ FGSCs exist in the ovarian cortex surface beneath the epithelium.^13^ Ovaries, the female reproductive organ, are both gonads and endocrine glands. They are the site of production and periodical release of egg cells. In our experiments, the OH‐supplied condition (condition 2) turned out to be insufficient for FGSC differentiation; instead, FGSCs retained better renewal and growth, suggesting that OH supports FGSC maintenance rather than initiation of differentiation. Therefore, we subsequently evaluated the synergistic effect of RA induction and OH conditioning (condition 3) and observed a more advanced differentiation status. Notably, differentiating FGSCs could be maintained for a remarkably longer period of time in condition 3.

Although morphological characteristics suggested differentiation of FGSCs into oocytes, differentiated cells could not produce more mature oocytes under condition 3. This may be ascribed to the fact that they failed to receive effective hormone stimulation. Oestrogen (E2) and progesterone (P4) are important endocrine hormones that regulate oocyte maturation.^28^ Thus, we next attempted to expose differentiating cells to condition 4 after culture with differentiation condition 3 for 10 days. When E2 and P4 stimulation was applied, differentiating FGSCs showed no significant changes compared with their unsupplemented counterparts. The differentiation process was further impeded by intensive cell apoptosis in prolonged culture, suggesting that E2 and P4 were not sufficient for the maturation of differentiating FGSCs; this might require more complex “niche” conditions.

Based on the above results, we further improved differentiation conditions. Granulosa cells transduce multiple signals to participate in initiation of oocyte meiosis and oocyte maturation through gap junctions and paracrine mechanisms. GV oocytes were obtained under differentiation condition 5, which included a hanging drop procedure and co‐culture of mFGSCs with granulosa cells. The progress of mFGSC commitment to differentiation was monitored by detecting the expression of meiosis‐specific marker *Scp3* and oocyte‐specific marker *Zp3*. 3D observation of IVD‐GVO revealed that both the nucleus‐plasma ratio and proportion of chromatin were not significantly different between IVD‐GVO and GVO, indicating in vitro differentiation GV oocytes from mFGSCs under this condition has a certain reliability. This result was consistent with previous studies performed by Zhou et al and Ding et al, who used a similar system to differentiate rat FGSCs and human FGSCs into GV‐stage oocytes in vitro*,* respectively.^18^, ^19^ This result indicates that this three‐step system is the most optimal differentiation condition for murine, rat and human FGSCs reported to date. Moreover, the in vitro differentiation mechanism of FGSCs may be conserved among species.

In summary, we evaluated five different in vitro differentiation conditions for mFGSCs and successfully differentiated mFGSCs into GV‐stage oocytes under a three‐step differentiation condition. To our knowledge, this is the first observation of mouse GV oocytes derived from mFGSCs in vitro. While further investigation is needed to determine whether it is possible to produce fertilizable oocytes from FGSCs in vitro, our study provides both a valuable model for studying the mechanisms underlying mammalian oogenesis and an important alternative source of oocytes.

## CONFLICT OF INTEREST

The authors declare that there is no conflict of interest regarding the publication of this article.
